# Pattern of Driver-Like Input onto Neurons of the Mouse Ventral Lateral Geniculate Nucleus

**DOI:** 10.1523/ENEURO.0386-22.2022

**Published:** 2023-01-17

**Authors:** Gubbi Govindaiah, Michael A. Fox, William Guido

**Affiliations:** 1 Department of Anatomical Sciences and Neurobiology, University of Louisville School of Medicine, Louisville, Kentucky 40202; 2 Center for Neurobiology Research, Fralin Biomedical Research Institute at Virginia Tech Carilion, Roanoke, Virginia 24016; 3 School of Neuroscience, Virginia Tech, Blacksburg, Virginia 24061

**Keywords:** corticothalamic, retinogeniculate, ventral lateral geniculate nucleus

## Abstract

The ventral lateral geniculate nucleus (vLGN) is a retinorecipient region of thalamus that contributes to a number of complex visual behaviors. Retinal axons that target vLGN terminate exclusively in the external subdivision (vLGNe), which is also transcriptionally and cytoarchitectonically distinct from the internal subdivision (vLGNi). While recent studies shed light on the cell types and efferent projections of vLGNe and vLGNi, we have a crude understanding of the source and nature of the excitatory inputs driving postsynaptic activity in these regions. Here, we address this by conducting *in vitro* whole-cell recordings in acutely prepared thalamic slices and using electrical and optical stimulation techniques to examine the postsynaptic excitatory activity evoked by the activation of retinal or cortical layer V input onto neurons in vLGNe and vLGNi. Activation of retinal afferents by electrical stimulation of optic tract or optical stimulation of retinal terminals resulted in robust driver-like excitatory activity in vLGNe. Optical activation of corticothalamic terminals from layer V resulted in similar driver-like activity in both vLGNe and vLGNi. Using a dual-color optogenetic approach, we found that many vLGNe neurons received convergent input from these two sources. Both individual pathways displayed similar driver-like properties, with corticothalamic stimulation leading to a stronger form of synaptic depression than retinogeniculate stimulation. We found no evidence of convergence in vLGNi, with neurons only responding to corticothalamic stimulation. These data provide insight into the influence of excitatory inputs to vLGN and reveal that only neurons in vLGNe receive convergent input from both sources.

## Significance Statement

The ventral lateral geniculate nucleus is traditionally thought of as a thalamic visual recipient structure. However, recent studies reveal its divergent output to a variety of nonvisual subcortical structures helps control an array of light-mediated defensive and mood-related behaviors. Despite this knowledge, we still lack an understanding of where and how inputs to this nucleus drive activity. Here we show that vLGN receives strong, driver-like excitatory input from two sources: the retina and cortical layer V. The external subdivision receives convergent input from both sources, whereas the internal division receives input only from layer V. Such an arrangement has important implications for understanding the functional organization of vLGN and its role in integrating vision with internal behavioral states.

## Introduction

Often mislabeled as a mere relay of sensory information, the visual thalamus is a complicated set of nearly a dozen nuclei, each with unique cell types, circuits, and functions (Monavarfeshani et al., 2017; Fratzl and Hofer, 2022). In nocturnal rodents, one of the largest of these nuclei is the ventral lateral geniculate nucleus (vLGN; Harrington, 1997). Despite its size, dense innervation by retinal ganglion cell (RGC) axons, and location adjacent to the well characterized dorsal lateral geniculate nucleus (dLGN), the neurons and circuits of the vLGN have only recently received attention. For example, studies reveal that vLGN is remarkably distinct from its dorsal counterpart in its transcriptome, proteome, cytoarchitecture, receptive field structure, and circuitry (Harrington, 1997; Su et al., 2011; Hammer et al., 2014; Monavarfeshani et al., 2017; Sabbagh et al., 2018, 2021; Ciftcioglu et al., 2020). While glutamatergic thalamocortical relay neurons are the predominant neuronal cell type in most thalamic nuclei including dLGN, virtually all neurons in vLGN are GABAergic, including their projection neurons (Gabbott and Bacon, 1994; Sabbagh et al., 2018, 2021; Huang et al., 2019, 2021; Salay and Huberman, 2021). These GABAergic neurons are richly diverse and highly organized, with transcriptionally distinct cell types stratified into laminar domains across the lateral–medial axis of the nucleus (Sabbagh et al., 2021). This organization has potential functional significance as axonal inputs, at least from the retina, innervate some regions of vLGN but not others. For example, neurons within the lateral-most external domain of vLGN (vLGNe), receive monosynaptic input from RGCs, whereas those in the medial, internal vLGN (vLGNi) do not appear to be retinorecipient (Hammer et al., 2014; Sabbagh et al., 2021; Beier et al., 2022). Indeed, these cell type-specific circuits are likely to underlie differences in the emerging functions of vLGN, which include light-mediated effects on depressive behavior, innate escape responses to visual threats (Huang et al., 2019; Fratzl et al., 2021; Huang et al., 2021; Salay and Huberman, 2021; Fratzl and Hofer, 2022), as well as oculomotor signaling and the photic regulation of circadian rhythms (Magnin et al., 1974; Harrington, 1997; Livingston and Fedder, 2003).

Although recent studies provide new details about the cellular composition, dendritic morphology, and visual receptive field properties of vLGN neurons (Ciftcioglu et al., 2020; Sabbagh et al., 2021), we still lack a detailed understanding of the full complement of inputs to this nucleus, information that would be key to better define the diverse functional repertoire of vLGN.

In the adjacent dorsal thalamus, identifying sources as “driver” or “modulator” input has led to a conceptual framework that delineates the functional organization of thalamic nuclei (Sherman and Guillery, 1998; Guillery and Sherman, 2002; Sherman and Guillery, 2002; Petrof and Sherman, 2013; Bickford, 2016; Usrey and Sherman, 2019). For example, first-order nuclei such as the dLGN receive “driver-like” inputs from the periphery or subcortical structures and convey this information to neocortex. In contrast, higher-order thalamic nuclei, such as the pulvinar, do not receive significant direct input from the periphery and instead receive driver-like input from cortical layer V neurons. Thus, higher-order thalamic nuclei serve as a conduit for cortico–cortico communication. Perhaps such distinctions may not be appropriate for vLGN, since it is developmentally distinct from dorsal thalamus, contains mostly GABAergic neurons, and does not project to neocortex. Nonetheless, it raises important questions about whether retinogeniculate (RG) and/or corticothalamic (CT) inputs function as drivers or modulators of activity in vLGN. Retinal axons innervating target neurons in vLGN differ significantly in structure and function from canonical retinogeniculate synapses in dLGN (Hammer et al., 2014). Neurons in vLGN have diffusely organized receptive fields and receive input from both image-forming and non-image-forming RGCs (Monavarfeshani et al., 2017; Ciftcioglu et al., 2020; Beier et al., 2022). However, they do exhibit some features associated with driver inputs, such as large ionotropic glutamatergic synaptic responses that exhibit depression following repetitive stimulation (Petrof and Sherman, 2013; Hammer et al., 2014; Sabbagh et al., 2021). Another difference between vLGN and dLGN is the source of corticothalamic innervation. vLGN lacks modulatory feedback from cortical layer VI, but instead receives input from layer V of cortex (Bourassa and Deschenes, 1995; Jacobs et al., 2007; Brooks et al., 2013; Seabrook et al., 2013), a source that provides driver-like input to high-order thalamic nuclei (Usrey and Sherman, 2019; Mease and Gonzalez, 2021). Thus, vLGN may receive driver-like input from both the retina and cortex.

To explore this possibility, we used optogenetic techniques to functionally characterize retinal and cortical inputs to neurons residing in vLGNe and vLGNi. By adopting a dual-opsin approach, we could independently examine in identified vLGN neurons the light-evoked synaptic responses arising from these two sources. Our results reveal that neurons in vLGNe receive convergent driver-like synaptic input from retinal ganglion cells and layer V cortical neurons. By contrast, neurons residing in vLGNi only receive driver-like input from layer V. These results provide a much-needed functional characterization of connectivity in vLGNe and vLGNi and offer initial evidence that these regions may in fact be distinct nuclei, rather than subdomains of a single nucleus.

## Materials and Methods

### Subjects

Experiments were conducted in adult mice (age, >30 d) of either sex. We crossed Tg(Rbp4-Cre) mice (KL100GSat/Mmcd, GENSAT RP24 -285K21) with the Rosa-CAG-LSL-ChR2(H134R)-EYFP-WPRE mice [commonly referred to as “Ai32,” which contains a loxP-flanked STOP cassette preventing the expression of a channel rhodopsin (ChR2)–enhanced yellow fluorescent protein (EYFP) fusion gene; stock #012569, The Jackson Laboratory; RRID:IMSR_JAX:012569) and used the offspring to examine postsynaptic responses of vLGN neurons evoked by blue light stimulation of corticothalamic layer V terminals. All breeding and experimental procedures were approved by the institution.

In total, we recorded 63 vLGNe and 21 vLGNi neurons from 15 Rbp4 × Ai32 mice, with 3–10 neurons per mouse. Of these 15 mice, 3 received binocular eye injections of an adeno-associated virus (AAV) containing ChrimsonR-tdtomato cassette (see below). One Rbp4-Cre mouse received a binocular eye injection of the anterograde tracer cholera toxin subunit b (CTB).

### Eye injections

For some mice, we made binocular intravitreal injections of the tracer CTB conjugated to Alexa Fluor 488 (Thermo Fisher Scientific) or an AAV containing a ChrimsonR-tdtomato cassette (AAV2/2.Syn-ChrimsonR.tdT; catalog #59171, Addgene; Jaubert-Miazza et al., 2005; Dilger et al., 2015). Mice were deeply anesthetized with ketamine/xylazine (8 mg/0.6 mg/ml:0.01 ml/g). The sclera was pierced with a sharp-tipped glass pipette, and excess vitreous was drained. Another pipette, filled with a 0.1–0.2% solution of CTB or AAV ChrimsonR tdTomato, was inserted into the hole made by the first pipette. The pipette containing solution was attached to a picospritzer and a prescribed volume (3–5 μl) of solution was injected into the eye. After a 2 d (CTB) or 14–21 d (AAV-CrimsonR-tdTomato) survival time, mice were killed and transcardially perfused with PBS and 4% paraformaldehyde, and brains were postfixed in 4% paraformaldehyde for 12 h. Fixed brains were cut in the coronal plane sectioned (80–100 μm) on a vibratome (catalog #1200S, Leica) and mounted in ProLong Gold (Thermo Fisher Scientific).

### Brain slice preparation

Animals were deeply anesthetized with isoflurane and decapitated, and the brains were transferred into cold (∼4°C), oxygenated (95% O_2_/5% CO_2_) slicing solution containing the following (in mm): 2.5 KCl, 26 NaHCO_3_, 1.25 NaH_2_PO_4_, 10.0 MgCl_2_, 2.0 CaCl_2_, 234.0 sucrose, and 11.0 glucose. Coronal brain slices (thickness, 270–300 μm) at the level of vLGN were cut using a vibrating tissue slicer (catalog #1200S, Leica). Slices were incubated in a holding chamber at 32°C for 30 min and then at room temperature in an oxygenated (95% O_2_/5% CO_2_) artificial CSF (ACSF) consisted of the following (in mm): 126.0 NaCl, 26.0 NaHCO_3_, 2.5 KCl, 1.25 NaH_2_PO_4_, 2.0 MgCl_2_, 2.0 CaCl_2_, and 10.0 glucose. Individual slices were then transferred to the recording chamber just before where whole-cell recordings were performed from neurons that were superfused with oxygenated ACSF at 3 ml/min and maintained at 32°C.

### Whole-cell recording procedures

**Figure 1.**
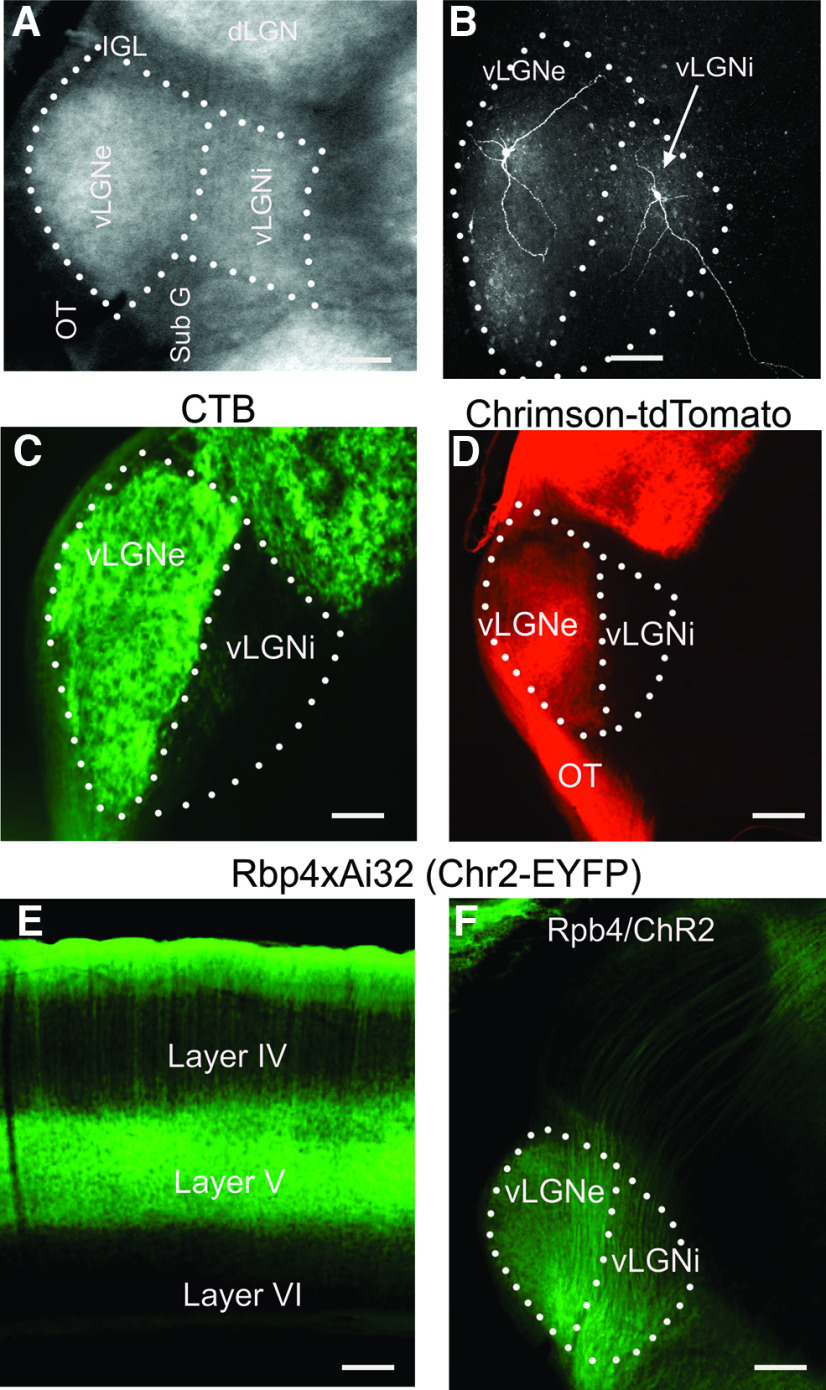
Subdivisions of the vLGN along with the pattern of projections from the retina and layer V of visual cortex. ***A***, DIC image of the acute thalamic slice preparation (270 μm, coronal plane) that was fixed overnight after *in vitro* recording. ***B***, *Z*-stack reconstructions of neurons located in the vLGNe and vLGNi that were filled with biocytin during *in vitro* recordings. ***C***, Coronal section through the dorsolateral thalamus showing the pattern of retinogeniculate axon terminal labeling after an intravitreal injection of CTB conjugated to Alexa Fluor 488 dye. Retinal terminal labeling is restricted to the vLGNe. ***D***, Coronal section through the dorsolateral thalamus showing the pattern of retinogeniculate axon and terminal labeling after an intravitreal injection of AAV containing the opsin ChrimsonR-tdTomato. Retinal terminal labeling is present in the vLGNe, IGL, and dLGN. Note the lack of labeling in vLGNi. ***E***, Coronal section through the visual cortex of an Rbp4 × Ai32 mouse depicting the expression of EYFP in layer V neurons. The cross of these mouse lines targets ChR2-EYFP in Cre-expressing neurons of layer V of neocortex. ***F***, Coronal section through dorsolateral thalamus showing the pattern of EYFP labeling of corticothalamic layer V axons coursing through dLGN, along with the selective terminal labeling in vLGNe and vLGNi. Note the lack of terminal labeling in dLGN. Scale bar, 100 μm.

Whole-cell recordings were obtained from neurons located in the external and internal subdivisions of vLGN (i.e., vLGNe and vLGNi; Govindaiah and Cox, 2009; Hammer et al., 2014; Sabbagh et al., 2021). Recordings were obtained with the visual aid of a microscope (model BX51W1, Olympus) equipped with differential interference contrast (DIC) optics where it was possible to distinguish external and internal subdivisions of vLGN (Fig. 1*A*). Borosilicate recording pipettes were pulled using a two-stage vertical puller (Narishige) and had tip resistances of 6–9 MΩ when filled with internal solution. To record excitatory postsynaptic currents and voltage (EPSC/EPSP), the internal recording solution contained the following (in mm): 117.0 K-gluconate, 13.0 KCl, 1.0 MgCl_2_, 0.07 CaCl_2_, 0.1 EGTA, 10.0 HEPES, 2.0 Na-ATP, 0.4 Na-GTP, and 0.3% biocytin. The pH and osmolarity of internal solution were adjusted to 7.3 and 290 mOsm, respectively. After forming the whole-cell configuration, the recording was allowed to stabilize for ≥2 min before data acquisition. Voltage-clamp recordings were performed at a holding potential of −70 mV, whereas current-clamp recordings were obtained at the resting membrane potential (−62 to 64 mV). Pipette capacitance, series resistance, input resistance, and whole-cell capacitance were monitored throughout the recording session. Recordings were conducted in neurons with resting membrane potentials between −55 and −75 mV, overshooting action potentials, and series resistance between 10 and 25 MΩ. Only recordings in which series resistance remained relatively stable (<20% change) were included in the analysis (Govindaiah and Cox, 2009; Govindaiah et al., 2012, 2020; Jurgens et al., 2012; Sokhadze et al., 2019). Signals were amplified using an amplifier (model Multiclamp 700B, Molecular Devices) and subsequently digitized at 2.5–5 kHz, low-pass filtered at 10 kHz, and stored on computer for offline analyses using pClamp software.

### Synaptic stimulation

To electrically evoke postsynaptic retinogeniculate synaptic activity in vLGNe neurons, square-wave pulses (0–200 μA, 1 ms duration, 10 pulses of 1–50 Hz) were delivered through a tungsten bipolar microelectrode (FHC) positioned in the optic tract (OT) near the targeted structure (Govindaiah and Cox, 2009; Hammer et al., 2014; Sabbagh et al., 2021). For photoactivation of corticothalamic terminals we used a blue light (460 nm)-emitting diode (model UHP 460, Prizmatix), and for retinogeniculate terminals, a red one (630 nm) that were delivered through a 60× objective. This produced a spot of light at the preferred wavelength onto the submerged slice with an approximate diameter of 0.3 mm. Pulse duration and frequency were controlled using pClamp software (Molecular Devices). For repetitive stimulation, 1 ms pulses were delivered at different temporal frequencies (10 pulses of 1–20 Hz). Most of the experiments were performed using an intensity of 95–112 mW/mm^2^. In some cases (see Fig. 3*F*,*G*), where we examined the degree of corticothalamic convergence (see Fig. 3*F*), recordings were obtained by varying a single pulse of light at an intensity between 0% and 100% of maximal intensity (460 nm = 112 mW/mm^2^; 630 nm = 95 mW/mm^2^) in 10% incremental steps.

**Figure 2.**
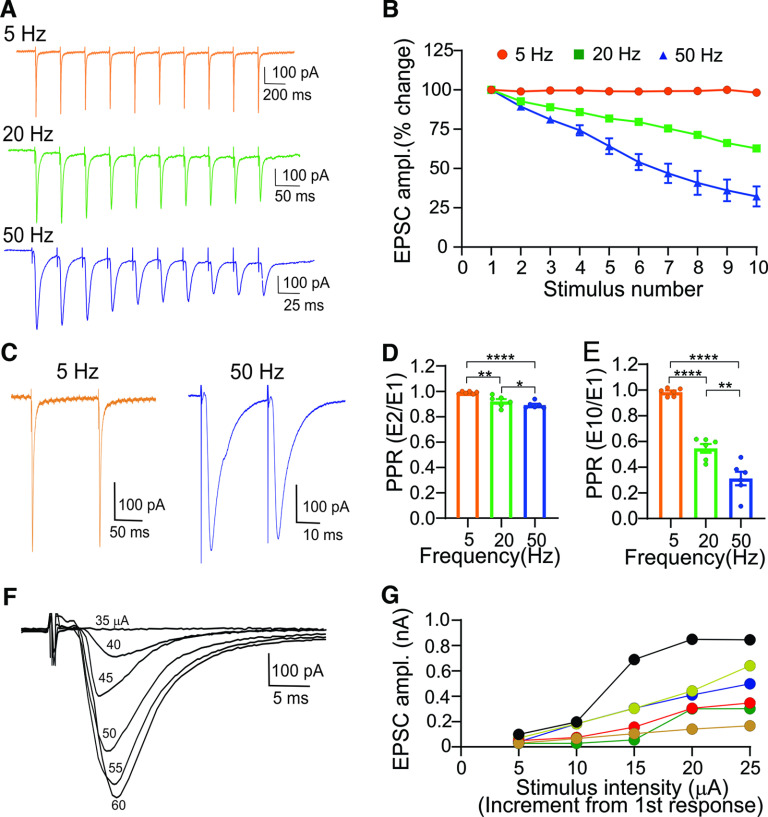
Electrical stimulation of the OT evokes EPSCs in vLGNe. ***A***, Whole-cell voltage-clamp recordings from a vLGNe neuron showing EPSCs evoked by repetitive OT stimulation at 5, 20, and 50 Hz. EPSCs display frequency-dependent depression of EPSC amplitude. Stimulus artifacts in the traces depict the timing of OT stimulation. ***B***, Summary graph (*n *=* *6 neurons) showing the changes in EPSC amplitude as a function of stimulus number for trains of 5, 20, and 50 Hz. Changes in EPSC amplitude are expressed as the percentage change from the initial response. Symbols depict mean ± SEM ***C***, Examples of EPSCs that exhibit paired pulse depression at 5 and 50 Hz (EPSC_1_ vs EPSC_2_). ***D***, ***E***, Summary graphs of PPRs for trains of 5, 20, and 50 Hz. PPRs are expressed as the ratio of the initial EPSC compared with the 2nd (***D***; EPSC_2_/EPSC_1_) or 10th (***E***; EPSC_10_/EPSC_1_) response. Bars reflect the mean ± SEM, and symbols reflect individual values. Asterisks depict significant differences based on Tukey’s multiple-comparisons test (***E***; PPR E_2_/E_1_: ****5 vs 50 Hz, *p *<* *0.0001; **5 vs 20 Hz, *p *=* *0.009; *20 Hz vs 50 Hz, *p *=* *0.04; ***F***, PPRs E_10_/E_2_: ****5 vs 50 Hz, *p *<* *0.0001; **5 vs 20 Hz, *p *<* *0.0001; **20 vs 50 Hz, *p *=* *0.001). ***F***, Example of graded synaptic response for a vLGNe neuron evoked by a progressive increase in stimulus intensity to OT stimulation. EPSCs are superimposed, and numbers reflect stimulus intensity (μA). ***G***, Input–output functions for 6 neurons illustrating the increase in EPSC evoked by progressive increase in stimulus intensity. Symbols reflect individual values, and each function represents a single neuron. Stimulus intensity is expressed in 5 μA increments from a subthreshold value recorded at baseline.

**Figure 3.**
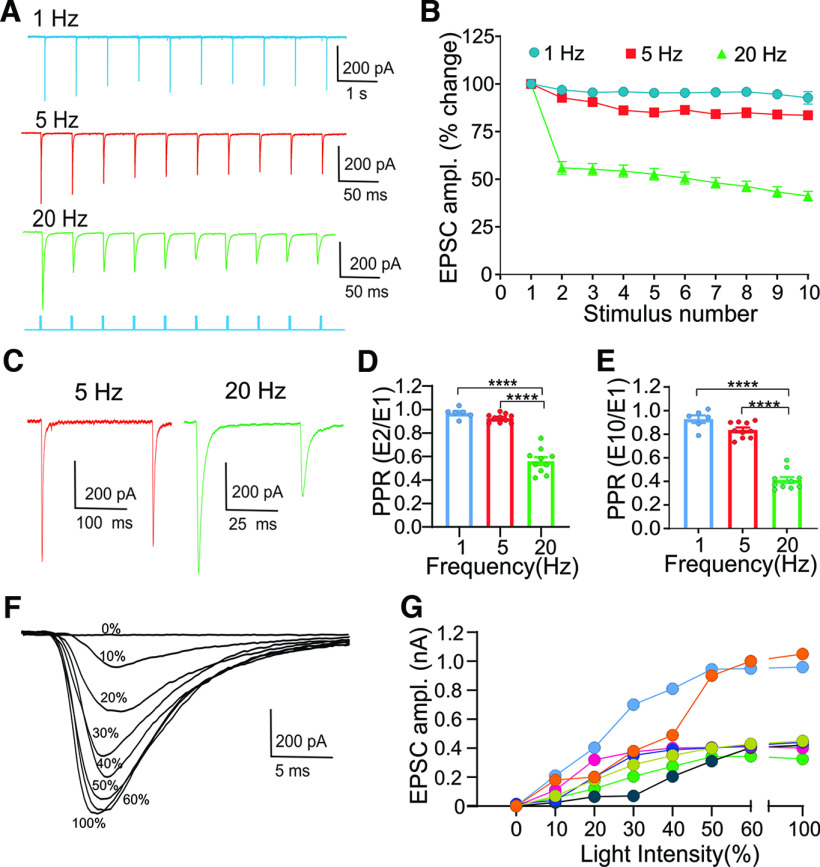
Photostimulation of layer V cortical terminals evokes EPSCs in vLGNe neurons in the Rbp4 × Ai32 (ChR2-EYFP) mouse. ***A***, Whole-cell voltage-clamp recordings from a vLGNe neuron showing EPSCs evoked by repetitive blue light stimulation (blue bars beneath the traces) at different temporal frequencies (1 ms pulse at 1, 5, and 20 Hz) of ChR2-expressing layer V corticothalamic terminals. EPSCs display frequency-dependent depression of EPSC amplitude. ***B***, Summary graph vLGNe neurons showing the changes in EPSC amplitude as a function of stimulus number for trains of 1 Hz (*n *=* *6 neurons), 5 Hz (*n *=* *10), and 20 Hz (*n *=* *10). Changes in EPSC amplitude are expressed as the percentage change of the initial response. Symbols depict the mean ± SEM. ***C***, Whole-cell voltage-clamp recordings from a vLGNe neuron showing paired-pulse depression for EPSC_1_ and EPSC_2_ at 5 and 20 Hz. ***D***, ***E***, Summary graphs of PPRs for trains of 1, 5, and 20 Hz. PPRs are expressed as the ratio of the initial EPSC compared with the 2nd (***D***; EPSC_2_/EPSC_1_) or 10th (***E***; EPSC_10_/EPSC_1_) response. Bars depict the mean ± SEM, and symbols depict individual values. Asterisks reflect significant differences, based on Tukey’s multiple-comparisons test (***D***; PPR E_2_/E_1_: ****1 vs 20 Hz, *p *<* *0.0001**;** ****5 vs 20 Hz, *p *<* *0.0001; ***E***, PPR E_10_/E1: ****1 vs 20 Hz, *p *<* *0.0001; ****5 vs 20 Hz, *p *<* *0.0001). ***F***, Example of graded EPSCs for a vLGNe neuron evoked by a progressive increase in blue light stimulus intensity. EPSCs are superimposed, and the number reflects the percentage of the maximal light intensity (112 mW/mm^2^). ***G***, Input–output functions for 6 neurons illustrating the increase in EPSC evoked by a progressive increase in stimulus light intensity. Symbols reflect individual values, and each function represents a single neuron. Stimulus intensity is expressed as a percentage increase in light intensity.

To examine the degree of retinal convergence (Fig. 2*F*) we generated EPSC amplitude by stimulus intensity plots (Seabrook et al., 2013; Hammer et al., 2014; Dilger et al., 2015; Tschetter et al., 2018). These were constructed by first determining the minimum stimulus intensity (average +/– SD) needed to evoke a postsynaptic response for at least three of five trials (typically 2 SDs above rms value for peak-to-peak noise). Once determined, current intensity was increased in small increments (5 μA) until a response of maximal amplitude was consistently reached. For each stimulus intensity, three to five responses were obtained. To examine the degree of corticothalamic convergence (Fig. 3*F*), we adopted a similar protocol for the photoactivation of corticothalamic terminals, in which the intensity to a single pulse of blue light (1 ms) was increased in 10% increments.

### Histology

To recover the morphology of neurons and verify their location within vLGN, biocytin (0.3% w/v; Sigma-Aldrich) was included in the recoding electrode and allowed to diffuse into the cell during recording using methods described previously (Krahe et al., 2011; El-Danaf et al., 2015; Charalambakis et al., 2019; Campbell et al., 2020). Following the recording sessions, slices were placed in a fixative consisting of 4% paraformaldehyde in PBS overnight, then washed in PBS, and treated with 0.1% Triton X-100 and Alexa Fluor 647-conjugated strepavadin (1 : 1000; catalog #S21374, Thermo Fisher Scientific) in PBS for 24 h. Slices were then rinsed with PBS, mounted with Prolong Gold (catalog #P36930, Thermo Fisher Scientific), and coverslipped for digital imagery. Images of biocytin-filled neurons were acquired using a multiphoton laser-scanning confocal microscope (model FV1200BX61, Olympus).

### Data analyses

The synaptic responses evoked by electrical photostimulation were measured using pClamp software (Molecular Devices). The amplitude of EPSC was measured from baseline values just before optic tract and/or photostimulation. The average of three to five trials was taken for measurements for each condition. All traces reflect the averaged responses of individual trials. To examine the changes in the synaptic response to repetitive stimulation, the percentage change in the EPSC amplitude was calculated from the initial response. To determine the degree of synaptic depression, paired-pulse ratios (PPRs) were obtained by calculating the EPSC amplitude of the 2nd or 10th response and dividing by the amplitude of the initial EPSC. Summary graphs include individual data along with mean ± SEM values. Statistical tests and levels of significance are provided in the Results. All *post hoc* comparisons and levels of significance indicated by asterisks are listed in the figure legends.

## Results

### Organization of vLGN

Figure 1 depicts the organization of vLGN, along with the main features of our experimental preparation, and the pattern of retinal and corticothalamic innervation. In acutely prepared thalamic slices cut in the coronal plane, the external and internal subdivisions of vLGN can be readily distinguished from each other and adjacent nuclei (Fig. 1*A*). By using biocytin**-**filled electrodes during our whole-cell *in vitro* recordings, we can target and confirm the location of neurons in each subdivision (Fig. 1*B*). The pattern of retinal innervation in vLGN is illustrated by making intravitreal injections of anterograde tracers into both eyes (Fig. 1*C*,*D*). Eye injections of CTB-Alexa Fluor 488 (Fig. 1*C*) or an AAV viral vector containing the channel rhodopsin variant ChrimsonR-td-Tomato (Fig. 1*D*) led to robust terminal labeling throughout the vLGNe, the intergeniculate leaflet (IGL), and the dLGN. The vLGNi was devoid of retinal terminals. To illustrate the pattern of corticothalamic innervation originating from layer V, we crossed RPB4-Cre mice with the Ai32 line to target ChR2-EYFP in Cre-expressing neurons of layer V (Fig. 1*E*). EYFP**-**labeled axons and terminal fields were present throughout both vLGNe and VLGNi, while axons could be seen coursing through dLGN and innervating the pulvinar (Fig. 1*F*).

Several laboratories make use of the Rpb4-Cre line to visualize or interrogate layer V corticothalamic projections, especially when done in conjunction with a Cre-dependent viral injection approach (Grant et al., 2016; Hoerder-Suabedissen et al., 2019; Prasad et al., 2020; Hayashi et al., 2021; Carroll et al., 2022; Casas-Torremocha et al., 2022). While the latter is ideal for achieving cell type specificity, it falls short in providing widespread and sufficient expression in a target structure, a task more readily achieved by using Cre-driver lines. Thus, we chose to take a Cre-driver approach rather than a Cre-viral vector one to maximize the expression of ChR2 in an acute thalamic slice preparation. As a result, it opens the possibility that off-target projections could contribute to our results. According to transcriptomic expression databases (Lein et al., 2007), *Rpb4*, the gene we use to drive Cre expression in layer V cells, is largely absent in mouse thalamus but is sparsely expressed in select regions of the midbrain (e.g., substantia nigra, superior colliculus, periaqueductal gray) and the brainstem (e.g., red nucleus). To our knowledge, most of these structures do not project to the vLGN (Harrington, 1997; Monavarfeshani et al., 2017; Fratzl and Hofer, 2022). However, the superior colliculus appears to project to the vLGN, at least in rodents and cats (Hughes and Chi, 1981; Pasquier and Villar, 1982; Cosenza and Moore, 1984; Fratzl et al., 2021). Thus, it is conceivable that Rbp4**-**expressing neurons from these regions comprise a fraction of ChR2**-**containing terminals in vLGN. It is important to note that Rpb4 expression in these off-target regions is very sparse and not well characterized. Moreover, it is unclear whether signaling arising from these neurons is excitatory or inhibitory, or whether they remain viable in an acute thalamic slice preparation. Thus, while these off-target inputs are potential contributors, they would appear to represent a small fraction of any of the excitatory responses evoked by stimulating layer V terminals. The most parsimonious explanation is that, compared with the massive layer V input to vLGN, these off-target inputs, while present, are of minimal impact.

### Electrical stimulation of the optic track in vLGNe

To examine retinogeniculate postsynaptic activity in vLGNe neurons, we used *in vitro* whole-cell recordings and electrical stimulation of optic tract fibers (Fig. 2; Hammer et al., 2014). Repetitive OT stimulation at different temporal frequencies (10 pulses at 5, 20, and 50 Hz) evoked trains of EPSCs that followed the temporal rate of stimulation (*n *=* *6 neurons). While the amplitude of EPSCs remained relatively stable at 5 Hz, increases in the temporal rate of stimulation to 20 and 50 Hz led to a decrease in amplitude with each successive stimulus pulse (Fig. 2*A*,*B*; ANOVA: *F*_(2,27)_ = 15.03, *p *<* *0.0001). The magnitude of synaptic depression was determined by calculating PPRs in which the amplitude of the initial response was compared with the 2nd response (EPSC_2_/EPSC_1_) or 10th response (EPSC_10_/EPSC_1_; Fig. 2*C*,*D*). PPRs based on EPSC_2_/EPSC_1_ (Fig. 2*D*) showed modest changes (mean ± SEM: 5 Hz, 0.98 ± 0.03; 20 Hz, 0.92 ± 0.01; 50 Hz, 0.89 ± 0.01), with PPRs at 20 and 50 Hz showing significant decreases compared with 5 Hz, and with those at 50 Hz showing the greatest change (ANOVA: *F*_(2,33)_ =16.25, *p *<* *0.0001). PPRs based on EPSC_10_/EPSC_1_ (Fig. 2*E*) showed greater depression (mean ± SEM: 5 Hz, 0.98 ± 0.01; 20 Hz, 0.54 ± 0.03; 50 Hz, 0.31 ± 0.05) with values at 20 and 50 Hz significantly <5 Hz (ANOVA: *F*_(2,33)_ = 84.08, *p *<* *0.0001).

We also examined the degree of retinal convergence onto vLGNe neurons by measuring the changes in EPSC amplitude evoked by increases (5 μA increments) in stimulus intensity. A systematic increase in stimulus intensity led to a progressive increase in EPSC amplitude (Fig. 2*F*; see also Hammer et al., 2014). Input–output curves (*n *=* *6 neurons) generated from these responses reflected a graded increase in amplitude as a function of stimulus intensity (Fig. 2*G*) and suggest that vLGNe neurons receive multiple retinal inputs (Chen and Regehr, 2000; Hammer et al., 2014; Dilger et al., 2015; Tschetter et al., 2018).

### Optogenetic activation of corticothalamic layer V terminals in vLGNe

To examine the synaptic responses associated with corticothalamic layer V input to vLGNe, we adopted an optogenetic approach. We crossed Rbp4 mice with the Ai32 line to express ChR2-EYFP in Cre**-**containing layer V neurons (Fig. 1*E*,*F*) and used blue light (460 nm) stimulation to photoactivate their terminals in thalamic slices while recording postsynaptic excitatory activity of vLGNe neurons (Fig. 3*A*). In 10 of 18 vLGNe neurons, repetitive stimulation (1, 5, and 20 Hz; 10 pulses at 1 ms) evoked large EPSCs (142–659 pA). When temporal rates >5 Hz were used, the amplitude of EPSCs began to attenuate after the initial pulse (Fig. 3*A*,*B*). At 1 Hz (*n *=* *6 neurons), little if any change in amplitude occurred throughout the entire stimulus train. While modest decreases were evident at 5 Hz (*n *=* *10 neurons), light delivery at 20 Hz (*n *=* *10 neurons) led to a substantial decline after the second pulse that remained stable throughout the duration of the stimulus train (ANOVA: *F*_(2,27)_ = 45.99, *p *=* *0.0001). PPRs based on EPSC_2_/EPSC_1_ (Fig. 3*C*,*D*) showed relatively small changes at 1 and 5 Hz (mean ± SEM: 1 Hz, 0.97 ± 0.01; 5 Hz, 0.92 ± 0.01), with PPRs at 20 Hz (0.56 ± 0.03) showing a significant decrease compared with 5 and 20 Hz (ANOVA: *F*_(2,23)_ = 87.48, *p *=* *0.0001). PPRs based on EPSC_10_/EPSC_1_ (Fig. 3*E*) showed a similar pattern (mean ± SEM: 1 Hz, 0.920 + 0.30; 5 Hz, 0.83 + 0.02; 20 Hz, 0.41 + 0.02) with values at 20 Hz significantly <1 and 5 Hz (ANOVA: *F*_(2,23)_ = 117.1, *p *=* *0.0001).

We also examined the responses to single blue light pulses (1 ms) in which the intensity of blue light stimulation was increased in 10% increments (0–100%). A systematic increase in stimulus intensity led to a progressive increase in EPSC amplitude (Fig. 3*F*). The input–output curves (*n *=* *7 neurons) generated from these responses reflected a graded increase in amplitude as a function of stimulus intensity (Fig. 3*G*). Such a profile suggests that vLGNe neurons receive multiple corticothalamic inputs.

### Dual-color opsin activation of retinogeniculate and corticothalamic terminals in vLGNe

**Figure 4.**
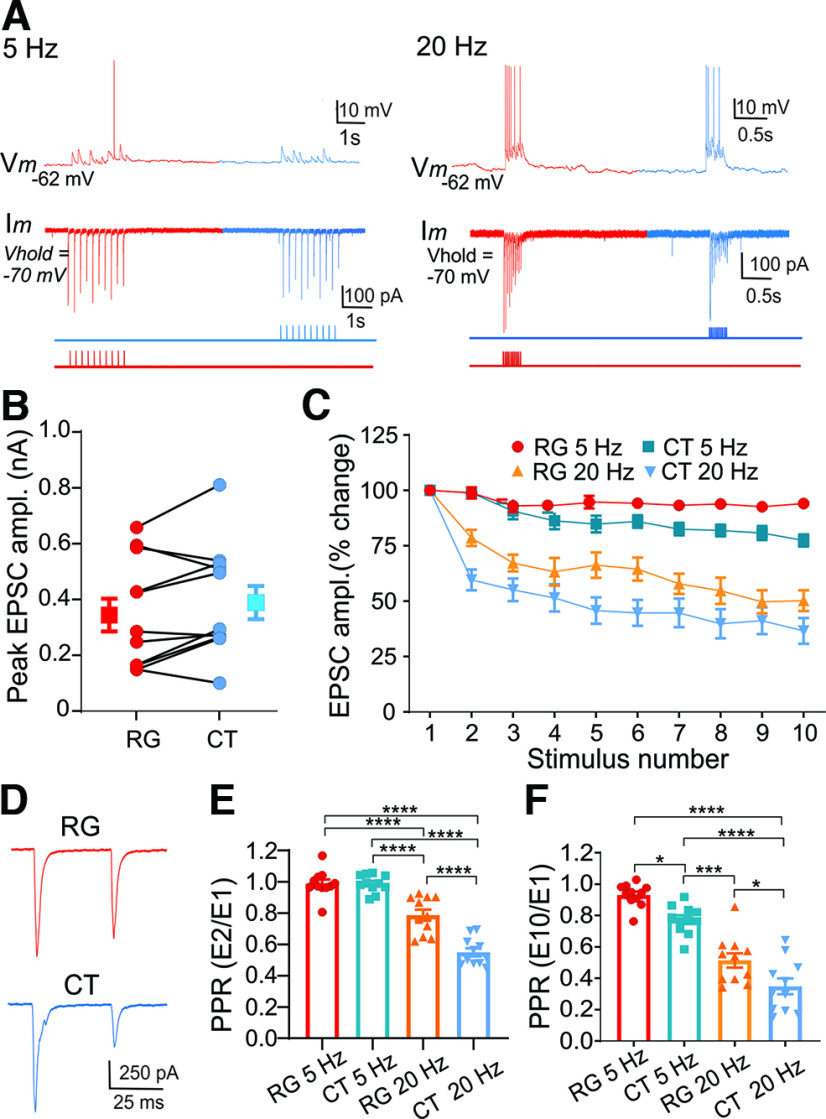
Dual-color opsin stimulation of retinogeniculate and corticothalamic terminals in vLGNe of the Rpb4 × Ai32 (ChR2-EYFP) mouse. ***A***, Intravitreal AAV injections of ChrimsonR-tdTomato led to red-shifted opsin expression in retinogeniculate terminals. Examples of whole-cell current-clamp (top trace) and voltage-clamp (bottom trace) recordings from the same vLGNe neuron showing the excitatory postsynaptic responses evoked by repetitive (5 and 20 Hz) red light (630 nm) stimulation of RG terminals with ChrimsonR (red traces) and blue light (460 nm) stimulation of CT terminals with ChR2 (blue traces). Red and blue bars beneath the traces correspond to the timing and sequence of photostimulation. ***B***, Graph showing peak amplitudes of EPSCs evoked by RG (red) and CT (blue) stimulation. Each pair of connected round symbols depicts a neuron (*n* = 11), and the large square symbols reflect the mean ± SEM. ***C***, Summary graph for vLGNe neurons showing the changes in EPSC amplitude as a function of stimulus number for trains of red light (RG) and blue light (CT) stimulation presented at 5 and 20 Hz. Changes in EPSC amplitude are expressed as the percentage change of the initial response. Symbols depict the mean ± SEM. Both retinogeniculate and corticothalamic stimulation lead to frequency-dependent depression. ***D***, Whole-cell voltage-clamp recordings from a vLGNe neuron showing paired-pulse depression (EPSC_1_ vs EPSC_2_) at 20 Hz after RG and CT stimulation. ***E***, ***F***, Summary graphs of PPRs after RG and CT stimulation for trains delivered at 5 and 20 Hz. PPRs are expressed as the ratio of the initial EPSC compared with the 2nd (***E***; EPSC_2_/EPSC_1_) or 10th (***F***; EPSC_10_/EPSC_1_) response. Bars reflect the mean ± SEM, and symbols reflect individual values. Asterisks depict significant differences based on Tukey’s multiple-comparisons test (E_2_/E_1_: ****RG 5 Hz vs CT 20 Hz, *p *<* *0.0001; ****RG 5 Hz vs RG 20 Hz, *p *<* *0.0001; ****CT 5 Hz vs CT 20 Hz, *p *<* *0.0001; ****RG 20 Hz vs CT 20 Hz, *p *=* *0.0001; PPR E_10_/E_1_: *RG 5 Hz vs CT 5 Hz, *p *=* *0.02; ****RG 5 Hz vs CT 20 Hz, *p *<* *0.0001; ****CT 5 Hz vs CT 20 Hz, *p *<* *0.0001; ***CT 5 Hz vs RG 20 Hz, *p *=* *0.0001; *RG 20 Hz vs CT 20 Hz, *p *=* *0.02).

To examine the convergence of retinogeniculate and corticothalamic inputs onto vLGNe neurons, we took a dual-color opsin approach that involved using a red-shifted opsin variant, ChrimsonR (620 nm), to activate retinal terminals, and ChR2 (460 nm), to activate corticothalamic terminals. To accomplish this, we made AAV eye injections of ChrimsonR-tdTomato in RPB4-Cre × Ai32 mice (Fig. 1*D*–*F*). For each neuron tested, repetitive pulses (1 ms, 10 pulses, 5 and 20 Hz) of red light (620 nm) and blue light (460 nm) were presented in a sequence (interstimulus intervals: 200 ms at 5 Hz; 50 ms at 20 Hz) as depicted in Figure 4*A*. Of 28 neurons tested, 11 appeared to receive convergent input from retinal and corticothalamic terminals, showing light-evoked excitatory postsynaptic activity to red (retina) and blue (corticothalamic) light stimulation (Fig. 4*A*). For those vLGNe neurons receiving convergent input, their peak responses were similar in strength, suggesting that these neurons receive equivalent excitatory input from both sources (Fig. 4*B*; mean ± SEM: retinogeniculate, 350 ± 59 pA; corticothalamic, 395 ± 60 pA; paired *t* test: *t *=* *1.812, df = 10, *p *=* *0.1001). As noted above, repetitive stimulation of retinal or corticothalamic terminals led to a frequency-dependent form of synaptic depression (Fig. 4*C*) with 20 Hz showing a greater degree of depression than 5 Hz (ANOVA: *F*_(3,32)_ = 92.4, *p *<* *0.0001). PPRs based on EPSC_2_/EPSC_1_ or EPSC_10_/EPSC_1_ further suggest that corticothalamic stimulation led to more depression than retinal stimulation (Fig. 4*D*–*F*). For example, at 20 Hz, EPSC_2_/EPSC_1_ ratios were significantly lower for corticothalamic than retinogeniculate stimulation (mean ± SEM: retinogeniculate at 20 Hz, 0.78 + 0.03; vs corticothalamic at 20 Hz, 0.50 + 02; ANOVA: *F*_(3,40)_ = 59.2, *p *<* *0.0001). A similar pattern emerged for PPRs based on EPSC_10_/EPSC_1_, with ratios associated with corticothalamic stimulation showing a greater amount of depression than those associated with retinogeniculate stimulation (mean ± SEM: retinogeniculate at 20 Hz, 0.52 + 0.04; vs corticothalamic at 20 Hz, 0.35 + 0.05; ANOVA: *F*_(3,40)_ = 46.3, *p *<* *0.0001).

**Figure 5.**
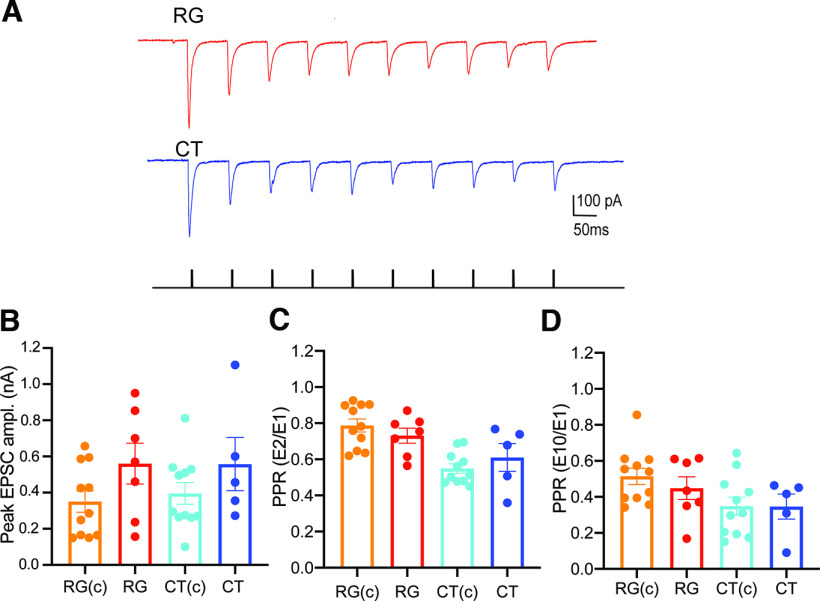
vLGNe neurons recorded under dual-color opsin stimulation that responded to a single excitatory source. ***A***, Examples of whole-cell voltage clamp from two vLGNe neurons, one (top) showing the excitatory postsynaptic responses evoked by repetitive (20 Hz) red light stimulation of RG terminals with ChrimsonR (red traces), and the other (bottom) by blue light stimulation of CT terminals with ChR2 (blue traces). Vertical black bars beneath the traces correspond to the timing of photostimulation. ***B–D***, Summary graphs comparing the peak responses (***B***) and PPRs (***C***, ***D***) of vLGNe neurons that exhibited convergent input [RG(c) and CT(c), *n* = 11; Fig. 4] with those that received input from a single source, either RG (*n* = 7) or CT (*n *=* *10). PPRs are expressed as the ratio of the initial EPSC compared with the 2nd (***C***; EPSC_2_/EPSC_1_) or 10th (***D***; EPSC_10_/EPSC_1_). Bars reflect the mean ± SEM, and symbols reflect individual values. Data for RG(c) and CT(c) are replotted from Figure 4*B–D* at 20 Hz. Values obtained from neurons that received convergent input were similar to those that received input from a single source.

In using the dual-color opsin approach, we found that not all vLGNe neurons showed evidence of convergent input. Of the 28 neurons tested, 7 responded only to red light (retinal) stimulation and 5 responded only to blue light (corticothalamic) stimulation, while 5 were unresponsive. The response profiles noted for vLGNe neurons that received retinogeniculate or corticothalamic input were similar to their convergent counterparts [RG(c) and CT(c)]. For example, RG or CT responses showed synaptic depression to 20 Hz repetitive light stimulation (Fig. 5*A*). Moreover, a comparison of peak amplitude (ANOVA: F_(3,30) = 1.60_, *p *=* *0.2093) and PPRs based on EPSC_2_/EPSC_1_ [RG(c) vs RG unpaired *t* test: *t *=* *1.005, df = 16, *p *=* *0.33; CT(c) vs CT unpaired *t* test: *t *=* *0.9568, df = 14, *p *=* *0.3549] or EPSC_10_/EPSC_1_ [RG(c) vs RG unpaired *t* test: *t *=* *0.8552, df = 16, *p *=* *0.4051; CT(c) vs CT unpaired *t* test: *t *=* *0.2425, df = 14, *p *=* *0.9810] showed values that were similar to those obtained from neurons receiving convergent input (Fig. 5*B–D*). Whether there are subpopulations of vLGNe neurons that receive driver-like input from a single source remains unclear since the lack of convergent input may simply be an artifact of the acute thalamic slice preparation where opsin-containing terminals are damaged or severed.

### Optogenetic activation of corticothalamic layer V terminals in vLGNi

**Figure 6.**
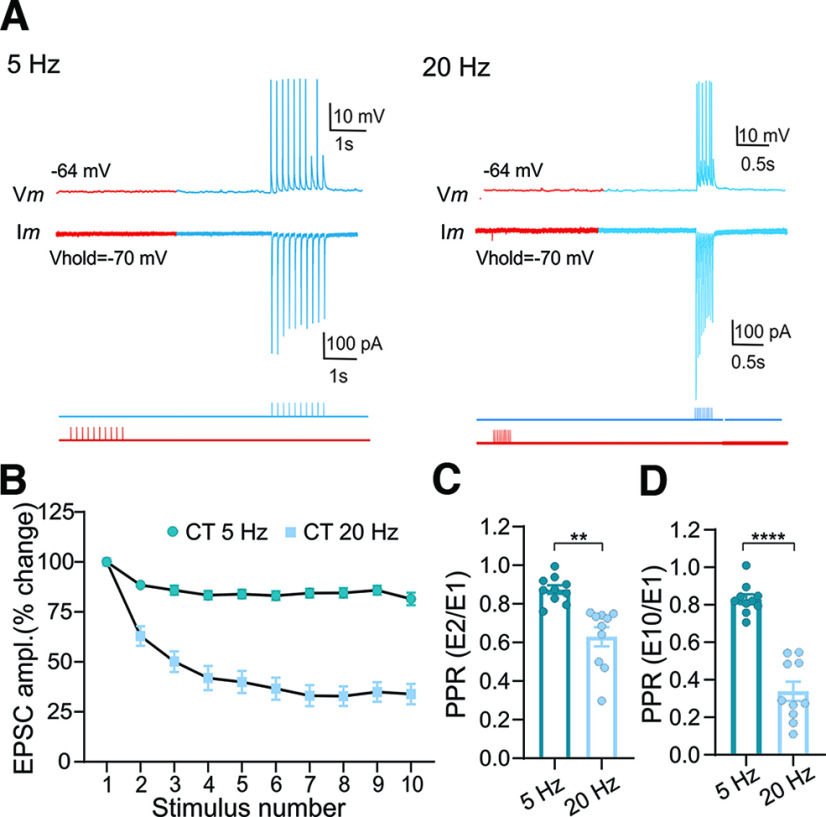
Dual-color opsin stimulation in vLGNi of the Rbp4 × Ai32 (ChR2-EYFP) mouse. Intravitreal AAV injections of ChrimsonR-tdTomato led to red-shifted opsin expression in retinogeniculate terminals. ***A***, Examples of whole-cell current-clamp (top trace) and voltage-clamp (bottom trace) recordings from a vLGNi neuron showing the excitatory postsynaptic responses evoked by repetitive blue light stimulation (5 and 20 Hz) of CT terminals with ChR2 (blue traces). Since vLGNi neurons are devoid of retinal input, red light stimulation fails to evoke a response (red traces). Red and blue bars beneath the traces correspond to the timing and sequence of photostimulation. ***B***, Summary graph for vLGNi neurons (*n *=* *10) showing the changes in EPSC amplitude as a function of stimulus number for trains of blue light (CT) stimulation presented at 5 and 20 Hz. Changes in EPSC amplitude are expressed as the percentage change of the initial response. Symbols depict the mean ± SEM. Corticothalamic stimulation leads to frequency-dependent depression. ***C***, ***D***, Summary graphs of PPRs after corticothalamic stimulation for trains delivered at 5 and 20 Hz. PPRs are expressed as the ratio of the initial EPSC compared with the 2nd (***C***, EPSC_2_/EPSC_1_) or 10th (***D***, EPSC_10_/EPSC_1_) response. Bars reflect the mean ± SEM, and symbols reflect individual values. Asterisks reflect significant differences (***C***, E_2_/E_1_: **5 vs 20 Hz, *p *=* *0002; ***D***, E_10_/E_1_: ****5 vs 20 Hz, *p *<* *0.0001).

To examine the synaptic responses associated with corticothalamic input to vLGNi, we again used Rbp4-Cre × Ai32 mice. We also made AAV eye injections of ChrimsonR-tdTomato in these mice to verify that vLGNi neurons are devoid of retinal input and that red light stimulation would not lead to retinally evoked activity. As expected, red light stimulation of vLGNi neurons failed to evoke any postsynaptic activity, whereas blue light stimulation of corticothalamic terminals led to excitatory postsynaptic activity in 10 of 21 neurons tested (Fig. 6*A*). Much like the responses observed in vLGNe neurons, repetitive stimulation (1 ms, 10 pulses, 5 and 20 Hz) of corticothalamic terminals in vLGNi neurons led to frequency-dependent synaptic depression (Fig. 6*B*; paired *t* test: *t *=* *7.7, df = 9, *p *<* *0.001). At 5 Hz, a modest decrease was observed between the 2nd and 10th pulse. However, at 20 Hz, EPSC amplitude showed a sharp decline between the second and fifth pulse, but then stabilized throughout the remainder of the stimulus train (Fig. 5*B*). PPRs based on EPSC_2_/EPSC_1_ or EPSC_10_/EPSC_1_ showed a similar pattern (Fig. 5*C*,*D*). For example, PPRs based on EPSC_2_/EPSC_1_ were significantly lower at 20 Hz compared with 5 Hz (mean ± SEM: 5 Hz, 0.87 ± 0.02; vs 20 Hz, 0.62 ± 0.04; paired *t* test: *t *=* *4.23, df = 9, *p *= 0.002). Additionally, PPRs based on EPSC_10_/EPSC_1_ showed a similar pattern, with values at 20 Hz being significantly <5 Hz (mean ± SEM: 5 Hz, 0.84 ± 0.02; vs 20 Hz, 0.33 ± 0.04; paired *t* test: *t *=* *8.7, df = 9, *p *<* *0.0001).

### Comparison of light-evoked corticothalamic EPSCs in vLGNe and vLGNi

**Figure 7.**
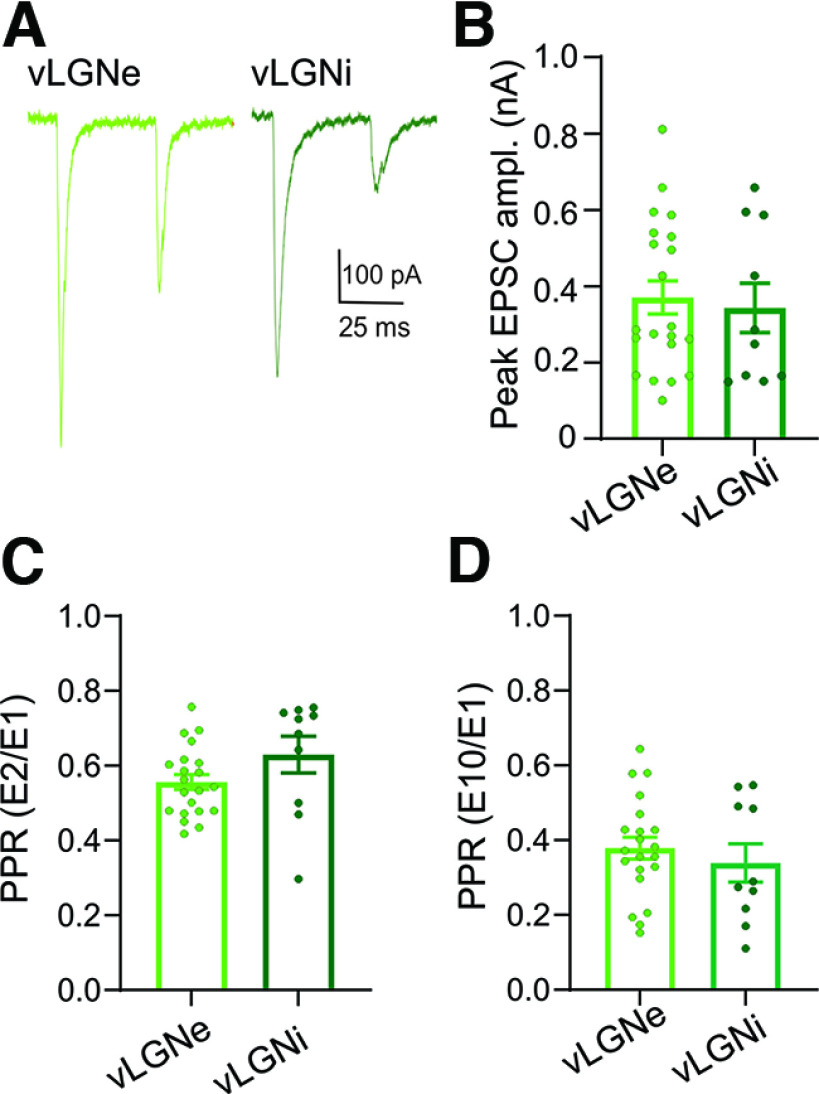
Comparison of synaptic responses evoked by blue light stimulation of corticothalamic terminals in vLGNe and vLGNi. ***A***, Whole-cell voltage-clamp recordings from a vLGNe (left) and vLGNi (right) neuron showing paired pulse depression (EPSC_1_ vs EPSC_2_) at 20 Hz after corticothalamic stimulation. ***B***, Summary graphs showing that peak responses for vLGNe (*n *=* *21; Figs. 3, 4) and vLGNi (*n *=* *10; Fig. 6) neurons are similar in magnitude. ***C***, ***D***, Summary graphs of PPRs after CT stimulation. PPRs are expressed as the ratio of the initial EPSC compared with the 2nd (***C***; EPSC_2_/EPSC_1_) or 10th (***D***, EPSC_10_/EPSC_1_). PPRs are similar for vLGNe and vLGNi neurons. For ***B–D***, bars reflect the mean ± SEM, and symbols reflect individual values.

A comparison of corticothalamic responses in vLGNe (*n *=* *21 neurons) and vLGNi (*n *=* *10 neurons) revealed similar profiles (Fig. 7*A*–*D*). For example, the peak EPSC amplitudes (Fig. 6*B*) for vLGNe and vLGNi neurons were comparable in magnitude (mean ± SEM: vLGNe, 371 ± 43 pA; vs vLGNi, 368 ± 59 pA; *t* test: *t *= 0.35, df= 29, *p *=* *0.7). Moreover, they displayed a similar form of synaptic depression when using repetitive (20 Hz) stimulation (Fig. 6*C*,*D*). PPRs based on EPSC_2_/EPSC_1_ (mean ± SEM: vLGNe, 0.55 ± 0.02; vs vLGNi, 0.62± 0.04; *t* test: *t *=* *1.6, df 29, *p *=* *0.1) or EPSC_10_/EPSC_1_ (mean ± SEM: vLGNe, 0.38 ± 0.03; vs vLGNi, 0.33 ± 0.05; *t* test: *t* = 0.7, df = 29, *p *=* *0.4).

### Potential interaction between ChrimsonR and ChR2 opsin crimson

**Figure 8.**
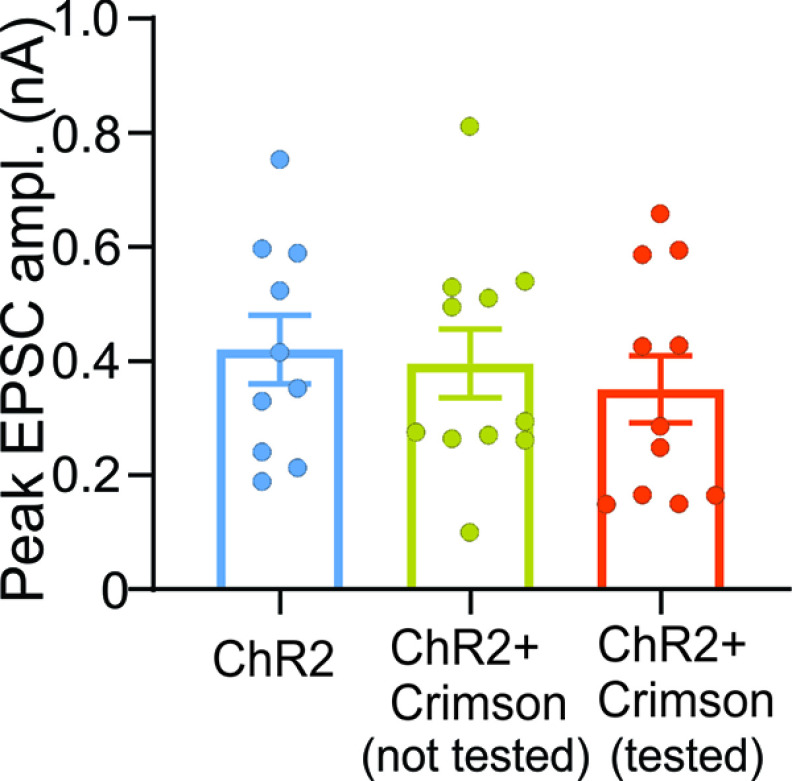
Comparison of peak EPSC amplitude for vLGNe neurons in the Rbp4 × Ai32 mouse (ChR2-EYFP) recorded under different opsin conditions. Summary graph showing the peak amplitude of EPSCs evoked with blue light stimulation of CT terminals with ChR2 (left, blue; ChR2, *n* = 10 neurons; Fig. 3), with blue light stimulation corticothalamic terminals with ChR2 in the presence of ChrimsonR in retinal terminals (middle, green; ChR2 ChrimsonR, *n* = 11) and with blue light stimulation of corticothalamic terminals with ChR2 and red light stimulation of retinal terminals with ChrimsonR (right, *n* = 11; Fig. 4). Bars reflect the mean ± SEM, and symbols reflect individual values. Note that peak amplitudes were similar across all conditions, suggesting that the blue light stimulation had little impact on ChrimsonR activation.

Although there is a large spectral separation between ChR2 and the red-shifted opsin CrimsonR, the latter still shows some (albeit weak) sensitivity to blue light stimulation (Klapoetke et al., 2014). Thus, ChrimsonR can be stimulated by red and blue light, while ChR2 is responsive only to blue light. As a result, for vLGNe neurons receiving convergent input, it is possible that the blue light stimulation, intended to target ChR2-containing corticothalamic terminals, could have inadvertently activated ChrimsonR-containing retinogeniculate terminals. To assess this, we compared the synaptic activity evoked across the following three different opsin conditions: blue light stimulation of corticothalamic terminals with ChR2 in the absence of ChrimsonR (*n *=* *10 neurons); blue light stimulation of corticothalamic terminals with ChR2 in the presence of CrimsonR in retinal terminals but not stimulated with red light (*n *=* *11 neurons); and blue light stimulation of corticothalamic terminals with ChR2 and red light stimulation of retinal terminals (*n *=* *10 neurons). A comparison of peak responses for vLGNe neurons under these stimulus conditions is shown in Figure 8. Indeed, peak amplitudes of EPSCs evoked by ChR2 alone (mean ± SEM, 420 ± 60 pA) were similar to the values obtained for ChR2-evoked responses recorded in the presence of ChrimsonR containing retinal terminals (mean ± SEM, 396 ± 60 pA), or when ChrimsonR-containing retinal terminals were activated by red light stimulation (mean ± SEM, 350 ± 59 pA; ANOVA: *F* = 0.34, df = 2,29, *p *=* *0.7). Together, these results suggest that the blue light stimulation had little impact on CrimsonR activation. Thus, for neurons receiving convergent input, the responses to blue light appeared driven exclusively by the activation corticothalamic terminals.

## Discussion

The past decade has seen a surge of interest in understanding the cells, circuits, and functions of the ventral lateral geniculate nucleus. Here we detail new information about the nature of the excitatory inputs that provide the primary drive for neurons within the external and internal divisions of vLGN. Using mouse transgenics, in conjunction with electrical and optical forms of stimulation to activate retinogeniculate or corticothalamic afferents, we show that vLGNe neurons receive strong convergent input from the retina and layer V of visual cortex. By contrast, vLGNi neurons receive input from visual cortex, but not from the retina. The synaptic responses evoked by retinal or corticothalamic stimulation appear driver like in nature, generating fast-rising, large-amplitude EPSC activity, and a frequency-dependent form of synaptic depression when repetitive stimulation is used (Guillery and Sherman, 2002; Li et al., 2003; Petrof and Sherman, 2013; Hammer et al., 2014).

Although some aspects of retinogeniculate input to vLGNe possess driver-like characteristics, they lack some of the hallmark features of drivers. For example, compared with retinogeniculate input to dLGN, vLGNe neurons showed somewhat weaker excitatory drive with high levels of retinal (and corticothalamic) convergence, as reflected in their input–output curves (Hammer et al., 2014), much larger visual receptive fields (Ciftcioglu et al., 2020), and smaller retinal terminals that tend to be located on more distal regions of dendrites (Hammer et al., 2014). Indeed, some of these features resemble modulator input (Petrof and Sherman, 2013), and, as suggested previously, retinogeniculate input to vLGNe may represent a hybrid form of input that does not strictly conform to the driver/modulator framework (Hammer et al., 2014; Bickford, 2016).

Similar to what is observed in higher-order nuclei such as LPN (lateral posterior nucleus) and POM (posterior medial nucleus) (Li et al., 2003; Reichova and Sherman, 2004; Groh et al., 2008), layer V input to both vLGNe and vLGNi was driver like, showing large, fast EPSC activity and strong frequency-dependent synaptic suppression. Based on immunohistochemistry staining with VGluTs in vLGNe, VGluT1-containing corticothalamic terminals appear similar in size to VGluT2-containing retinogeniculate terminals, which is in stark contrast to the large retinal terminals in dLGN (Hammer et al., 2014; Monavarfeshani et al., 2018). Moreover, they seem similar in size to those seen in higher-order nuclei that receive driver input from layer V (Rouiller and Welker, 2000; Groh et al., 2014; Prasad et al., 2020). In fact, the degree of synaptic depression associated with corticothalamic synapses in vLGNe was greater than observed with retinogeniculate synapses. Together, these observations suggest that corticothalamic input to vLGN should be considered as the more conventional or prototypical driver input.

It should be noted that while the convergence of driver-like inputs onto vLGNe is not a common feature of thalamic organization, it is not without precedence. For example, in dLGN, neurons within the shell subdivision receive driver-like input from the retina and SC (Bickford et al., 2015). In POM of somatosensory thalamus, single thalamocortical neurons receive driver-like input from the brainstem and cortical layer V (Groh et al., 2014). Overall, the significance of convergent driver-like input remains unclear, but the prevailing notion is that it allows for the interaction of complex cross-modal signaling between thalamus and other cortical or subcortical structures (Bickford, 2016; Mease and Gonzalez, 2021). Perhaps, in the case of vLGNe, it provides computational support for mediating visuomotor behavior associated with perceived fearful or threatening visual stimuli (Fratzl et al., 2021; Salay and Huberman, 2021; Fratzl and Hofer, 2022).

It is worth emphasizing that vLGNe is the exclusive site of convergence in vLGN. By contrast, vLGNi appears devoid of retinal input (Hickey and Spear, 1976; Harrington, 1997; Hammer et al., 2014; Monavarfeshani et al., 2017; Fig. 2) and, as our optogenetic experiments revealed, show no signs of retinally evoked activity. Such an arrangement adds to the growing list of features that distinguish vLGNe from vLGNi and further underscores the debate to consider vLGN as a single nucleus with divergent functions, or vLGNe and vLGNi as adjacent but distinct nuclei that have unique cell types and circuitry. Mounting evidence seems to support the latter. Clearly, vLGNe and vLGNi have distinct cytoarchitecture showing well delineated boundaries with vLGNe containing larger cells than vLGNi, features that led to delineating these regions as the magnocellular and parvocellular laminae of the vLGN (Niimi et al., 1963; Hickey and Spear, 1976; Gabbott and Bacon, 1994; Harrington, 1997). While the overwhelming majority of neurons in both regions of vLGN are GABAergic (Gabbott and Bacon, 1994; Yuge et al., 2011), traditional markers of neurochemically defined GABAergic neurons, such as parvalbumin-, calbindin-, and proenkephalin-expressing neurons, are differentially expressed in these regions with parvalbumin- and proenkephalin-expressing neurons residing exclusively in vLGNe and calbindin-expressing neurons residing in vLGNi (Sabbagh et al., 2021). Moreover, GABAergic interneurons labeled in the GAD67-GFP transgenic reporter line populate both vLGNe and dLGN but are absent from vLGNi (Su et al., 2011). Transcripts have also been identified that delineate vLGNe from vLGNi (Sabbagh et al., 2021) with *Neurexophilin-1* (*Nxph1*) mRNA being confined to vLGNe, whereas expression of the *Aristaless Related Homeobox* (*Arx*) gene is absent from vLGNe and enriched in vLGNi and other prethalamic nuclei. Furthermore, as one looks closely at the cellular landscape of vLGN, it is becoming clear that there may be differences in how cells are organized in each region. In vLGNe, neurons are organized into parallel, subtype-specific sublaminae, a feature that may play an important role in the processing of visual information (Beier et al., 2021; Sabbagh et al., 2021). At present, no such sublamina have been identified or characterized in vLGNi. Together, these data suggest that the transcriptome and cellular organization of vLGNi are more closely aligned with other, adjacent regions of the caudal prethalamus, such as zona incerta, than with vLGNe (Lein et al., 2007; Sabbagh et al., 2021). Indeed, along with vLGNi, a number of other inhibitory nuclei located in the caudal prethalamic region receive input from layer V, including the anterior pretectal nucleus, zona incerta, and the outer edge regions of thalamic reticular nucleus (Barthó et al., 2002; Bokor et al., 2005; Prasad et al., 2020; Carroll et al., 2022). Since these nuclei project to higher-order thalamic nuclei, input from layer V could serve to trigger a transthalamic inhibitory gating signal, perhaps activated during different behavioral states or in a modality-specific manner (Halassa and Acsády, 2016; Fratzl and Hofer, 2022).

Admittedly, we do not have a complete understanding of the afferent and efferent projections of vLGNe and vLGNi. As stated above, while both receive corticothalamic input, only vLGNe receives retinal input. Other anatomic studies suggest differences in brainstem cholinergic inputs, which are dense in vLGNe but sparse in vLGNi (Sokhadze et al., 2018, 2022). In the case of efferent projections, it is clear that vLGN projects to many subcortical visual and nonvisual targets, innervating nuclei in the hypothalamus, thalamus, midbrain, and brainstem (Harrington, 1997; Monavarfeshani et al., 2017; Huang et al., 2019, 2021; Fratzl et al., 2021; Salay and Huberman, 2021). However, it is unclear which of these projections arise from neurons in vLGNe and vLGNi.

Whether vLGNe or vLGNi are separate entities or simply subdivisions of a single nucleus, the hope is that with a deeper understanding of the organization of these regions, viral and genetic tools can be used to label and manipulate distinct populations of vLGN neurons to unravel differences in their connectivity and function.

## Synthesis

Reviewing Editor: Arvind Kumar, KTH Royal Institute of Technology Decisions are customarily a result of the Reviewing Editor and the peer reviewers coming together and discussing their recommendations until a consensus is reached. When revisions are invited, a fact-based synthesis statement explaining their decision and outlining what is needed to prepare a revision will be listed below. The following reviewer(s) agreed to reveal their identity: Jens Kremkow.

Synthesis

In this manuscript authors have characterized the structure of excitatory synaptic inputs to the external and internal subdivisions of the ventral lateral geniculate nucleus. The main focus is on the inputs from the retina and cortex. Authors show a convergence of drivers on single neurons, which is novel throughout thalamus and suggests vLGN serves as a site of receptive field integration.

Overall both the reviewers have appreciated the work but they also have raised their concerns such as:

At a number of places there is unclarity about experimental protocols and data displayed in figures. For details of this, please refer to the comments from the reviewers.

A more balanced reporting of the data and results is needed. For example, it appears that about 40% of the neurons in the vLGNe, and none in the vLGNi, actually receive convergent inputs. But this is not what the title suggests.

Besides these, reviewers have also raised a few more points that need to be addressed. Therefore, we would like to invite you to revise the manuscript and address the concerns of the reviewers in a point-by-point reply.

Reviewer #1

This neat manuscript establishes differences in retinal and cortical afferents between two divisions of vLGN, characterizes the physiological profiles of these afferents, and, where they overlap, uses a dual opsin approach to demonstrate convergence on single neurons. I believe the manuscript is acceptable for publication if the authors take additional care to clarify details of the experimental protocol, data analysis and clustering and certain technical limitations (primarily the use of the reporter line).

A pet peeve of this reviewer is a lack of explicit statements of aspects of experimental protocols in the literature. A few examples from this manuscript: 1) The authors seem to imply that minimum stimulation was employed; if so, state what criteria defined threshold- e.g. 50% likelihood of eliciting EPSPs of >5 mV? 2) Are the traces in your figures single or averaged? 3) Ns are reported in cells, but should also report clustering, i.e. number of animals. 4) How did authors ensure recorded cells were healthy i.e. a spike could be elicited, membrane resistance was monitored, etc.

As I understand it, some data is shared between 4 and 5. State clearly that Figure 4 only includes neurons receiving converging input, while Figure 5 includes all neurons. Perhaps use a different color in Figure 5C+D to indicate those neurons repeated from 4 to 5. Also to this point, did the authors test statistically whether the vLGNe cells receiving converging vs only one were drawn from the same population? A feasible hypothesis is that sole inputs might elicit a larger peak amplitude than converging inputs. Conversely, a slice preparation severs much of the circuitry, so some cases of non-convergence may represent false negatives.

Use of the Rbp4 reporter line is potentially problematic and bears some unpacking. The authors assert that labelled terminals in this line are derived from L5, which this reviewer challenges on a number of criteria. 1) Reporter lines are notoriously leaky (Prabhakar 2019) and 2) capture cells over development, when a Rbp4 phenotype might be transiently expressed among cells that lack it in maturity. 3) While Rbp4 is restricted to L5 in cortex, it is also expressed subcortically according to Allen ISH data, including sparsely in thalamus and brainstem. Whether these subcortical neurons are labelled in the reporter line is not shown in Fig 1E. 4) The Rbp4 reporter line has been widely used (e.g. by the Clasca lab) and these prior characterizations should be cited.

In the third paragraph of the introduction, the authors introduce layer 5 as a driver defining higher-order nuclei of dorsal thalamus. Inhibitory cells of the ventral thalamus should be included to strengthen this conceptual framework. A reference to cortico-cortico communication seems here out of place, as the authors note that vLGN includes inhibitory neurons of the ventral thalamus. Why not introduce here other cases of L5 input to inhibitory regions? L5 also inputs to APN (Bokor 2005, Prasad 2020), zona incerta, and the edge of TRN (Carroll 2021), which target higher-order thalamus. Perhaps these inhibitory regions along with vLGNi serve as a gating mechanism or cortico-thalamo-cortical pathways.

On the graded effect of EPSP amplitude with increasing stimulus intensity: Was the effect of intensity shown in Fig 2-3 F-G tested statistically (i.e. RM ANOVA)? Such a graded effect is associated with modulator inputs (e.g. Deschenes and Hu 1990), but this is not mentioned in the text. It might fit well in the 2nd and 3rd paragraph of discussion, where the authors list ways in which their synaptic measures deviate from the traditional driver phenotype.

A sentence fragment appears in the intro: “Whereas neurons residing in vLGNi....layer V”.

In the discussion’s fourth paragraph, the authors state that convergence of drivers is not a common feature of thalamic organization. Is this really known? Provide citations. The authors state that drivers from brainstem and layer 5 converge in POm but no citation is provided (I don’t believe this was shown at the level of single neurons, but rather of the nucleus overall).

Reviewer #2

In the study “Convergence of driver-like input onto neurons in the mouse ventral lateral geniculate nucleus” the authors investigate the synaptic inputs to neurons in the ventral lateral geniculate nucleus (vLGN) in vitro. The main focus of the study is to compare the properties of the synaptic inputs from the retina and cortex. In contrast to the dorsal LGN, little is known about these properties in the vLGN. I enjoyed reading the manuscript and the data and methods look solid. However, I do have a few comments that need to be addressed:

1) My main concern is about the overall picture that the manuscript conveys and the underlying supporting data. In the current version the manuscript gives the impression that convergence from retinal and cortical inputs is a common rule in the vLGN while in fact, it appears that only a small fraction of neurons in the vLGN receive both retinal and cortical inputs. Only neurons in the external domain of the vLGN (vLGNe), but not neurons in the internal domain (vLGNi), receive retinal and cortical inputs. Moreover, from those 28 neurons in the vLGNe only 11 receive convergent inputs from both sources. Thus, only about 40% of the neurons in the vLGNe, and none in the vLGNi, actually receive convergent inputs, which is in contrast to the title of the manuscript. Therefore, a more balanced reporting of the data/results is required in the title and main text.

2) Along this line: I think it could be helpful to include the data from the vLGNe neurons that do not receive convergent inputs but only from one source also in figure 4. At the moment figure 4 only shows data/examples from vLGNe neurons with converging inputs. Since the main aim of the figure is to compare RG and CT inputs this makes of course sense. However, I do think that showing also examples from neurons with only retinal and cortical inputs, together with some way of showing the fraction of neurons in each condition (pie chart?), could help the reader to better understand the data.

3) Similarly, only 10 out of 21 vLGNi neurons show synaptic activity upon light stimulation of the corticothalamic terminals. Where do the other ∼50% of vLGNi neurons get their inputs from? Answering this question is of course beyond the scope of this study, but conveying this information in the main text or figure appears to me to be important.

Minor:

1) Since the study focuses on two subdomains in the vLGN and two input sources it could be helpful to have a small cartoon in the figures showing from where the data is from and which pathway was tested. The figure legends contain this information, but adding a small cartoon/inset could make the figure/data even more approachable.

2) If the authors decide to keep “convergence” in the title it could be helpful to specify the sources (retinal, cortical) already in the title.

3) Figure 3A contains ticks for the optical stimuli, which looks nice. Maybe this could also look nice in Figure 2A?

## Author Response

Synthesis. At a number of places there is unclarity about experimental protocols and data displayed in figures. For details of this, please refer to the comments from the reviewers.

A more balanced reporting of the data and results is needed. For example, it appears that about 40% of the neurons in the vLGNe, and none in the vLGNi, actually receive convergent inputs. But this is not what the title suggests. Besides these, reviewers have also raised a few more points that need to be addressed. Therefore, we would like to invite you to revise the manuscript and address the concerns of the reviewers in a point-by-point reply.

Author Response. We addressed all concerns raised by the reviewers. As requested, we provide additional data in a new figure (Fig. 5) showing the synaptic responses of vLGNe neurons that receive input from a single source, either from corticothalamic or retinogeniculate projections. We also changed the title of the paper from “Convergence of driver-like input” to “Pattern of driver like input....” Below is a point-by-point response to reviewer concerns with the revisions highlighted in red.

Reviewer #1

R1. A pet peeve of this reviewer is a lack of explicit statements of aspects of experimental protocols in the literature. A few examples from this manuscript: 1) The authors seem to imply that minimum stimulation was employed; if so, state what criteria defined threshold- e.g. 50% likelihood of eliciting EPSPs of >5 mV? 2) Are the traces in your figures single or averaged? 3) Ns are reported in cells, but should also report clustering, i.e. number of animals. 4) How did authors ensure recorded cells were healthy i.e. a spike could be elicited, membrane resistance was monitored, etc.

Author response. Additional details pertaining to stimulation techniques, single or averaged traces, the number of neurons and animals, and neuronal health are addressed in the revision.

1. Synaptic stimulation. Our methods to determine the degree of retinal convergence are well established from previous work (Lo et al., 2002; Jaubert-Miazza et al.,2005; Ziburkus and Guido, 2006; Dilger et al., 2011; Seabrook et al., 2013; Hammer et al., 2014; Tschetter et al., 2018). For the current study we did not obtain estimates of the number of retinal inputs. Instead, we simply illustrated the relationship between EPSC amplitude and stimulus intensity (Hammer et al., 2014) by generating amplitude by stimulus intensity plots. Our studies and those of the Chen lab reveal that plots showing a gradual or graded increase in EPSP/EPSC amplitude reflect high levels of convergence, while those that are step-like (or all or none response profiles), reflect low levels of convergence.

(Methods and Materials, Synaptic stimulation) To examine the degree of retinal convergence (Fig. 2F) we generated EPSC amplitude by stimulus intensity plots (e.g., Seabrook et al., 2013; Hammer et al., 2014; Dilger et al., 2015; Tschetter et al., 2018). These were constructed by first determining the minimum stimulus intensity needed to evoke a postsynaptic response for at least 3 of 5 trials (typically two standard deviations above RMS value for peak-to-peak noise). Once determined, current intensity was increased in small increments (5uA) until a response of maximal amplitude was consistently reached. For each stimulus intensity, 3-5 responses were obtained. To examine the degree of corticothalamic convergence (Fig. 3F) we adopted a similar protocol for the photoactivation of corticothalamic terminals, in which the intensity to a single pulse of blue light (1ms) was increased in 10% increments.

2. Traces. (Methods and Materials, Data analysis). All traces reflect averaged responses of individual trials.

3. Number of animals and cells. (Methods and Materials, Subjects). In total, we recorded 63 vLGNe and 21 vLGNi neurons from 15 Rbp4 x Ai32 mice, with 3-10 neurons per mouse. Of these 15 mice, 3 received binocular eye injections of an AAV virus containing ChrimsonR-tdtomato cassette (see below). One Rbp4-Cre mouse received a binocular eye injection of the anterograde tracer CTB.

3. Neuronal Health. Our criteria for healthy, stable in vitro recordings have been published elsewhere (e.g., Govindaiah et al., 2009; Govindaiah et al., 2012; Jurgens et al., 2012; Sokhadze et al., 2019; Govindaiah et al., 2020) Reference to these citations were excluded in order to comply with the publisher’s request for anonymous author submission.

(Methods and Materials, Whole cell recording procedures). Pipette capacitance, series resistance, input resistance, and whole-cell capacitance were monitored throughout the recording session. Recordings were conducted in neurons with resting membrane potentials between -55 and -75 mV, overshooting action potentials, and series resistance between 10 and 25 MOhms. Only recordings in which series resistance remained relatively stable (<20% change) were included in the analysis (e.g., Govindaiah and Cox, 2009; Govindaiah et al., 2012; Jurgens et al., 2012; Sokhadze et al., 2019; Govindaiah et al., 2020).

R1. As I understand it, some data is shared between 4 and 5. State clearly that Figure 4 only includes neurons receiving converging input, while Figure 5 includes all neurons. Perhaps use a different color in Figure 5C+D to indicate those neurons repeated from 4 to 5. Also to this point, did the authors test statistically whether the vLGNe cells receiving converging vs only one were drawn from the same population? A feasible hypothesis is that sole inputs might elicit a larger peak amplitude than converging inputs. Conversely, a slice preparation severs much of the circuitry, so some cases of non-convergence may represent false negatives.

Author response.

The data in figure 4 pertains to vLGNe neurons that received convergent input, whereas the data in figure 5 refers to data from vLGNi. These two data sets are different populations; hence we are confused as to what the reviewer is requesting. Perhaps the reviewer was referring to Fig. 6 (Fig. 7 in revision) where we compare the corticothalamic responses of vLGNe and vLGNi neurons. Of the 21 vLGNe responses shown, 10 are from Fig. 3, and 11 are from Fig. 4 which plots the amplitudes of vLGNe neurons that received convergent input. This information is included in the revised figure legend. Additionally in the revision, we generated a new figure (Fig. 5) showing the responses of vLGNe neurons that did not receive convergent input, but instead received either retinogeniculate or corticothalamic input. We also provide an analysis comparing the peak amplitudes and PPRs of neurons that received convergent input (Fig. 4) with those that received input from one or the other source. Peak amplitudes and PPRs were comparable among all groups. It should be noted that lack of convergent input among vLGNe neurons could simply be an artifact of the slice preparation where inputs are damaged or severed. Such a possibility is a consequence of any study that employs an acute brain slice preparation.

(Results, Dual color opsin activation of retinogeniculate and corticothalamic terminals in vLGNe)

In using the dual color opsin approach, we found that not all vLGNe neurons showed evidence of convergent input. Of the 28 neurons tested, 7 responded only to red light (retinal) stimulation, 5 responded only to blue light (corticothalamic) stimulation, while 5 were unresponsive. The response profiles noted for vLGNe neurons that received retinogeniculate or corticothalamic input were similar to their convergent counterparts (RG(c) and CT(c)). For example, retinogeniculate (RG) or corticothalamic (CT) responses showed synaptic depression to 20Hz repetitive light stimulation (Fig. 5A). Moreover, a comparison of peak amplitude (ANOVA, F=1.60 (3,30),p=0.2093) and PPRs based on EPSC2/EPSC1 (RG(c) vs RG, unpaired t-test t=1.005 df=16, p=0.33; CT(c) vs CT unpaired t-test t=0.9568 df=14 p=0.3549) or EPSC10/EPSC1 (RG(c) vs RG unpaired t-test t=0.8552 df=16, p=0.4051; CT(c) vs CT unpaired t-test t=0.2425 df=14 p=0.9810) showed values that were a similar to those obtained from neurons receiving convergent input (Fig. 5B-D). Whether there are subpopulations of vLGNe neurons that receive driver-like input from a single source remains unclear, since the lack of convergent input may simply be an artifact of the acute thalamic slice preparation where opsin containing terminals are damaged or severed. Such a possibility is a consequence of any study that employs an acute brain slice preparation.

(Figure legends) Figure 5. vLGNe neurons recorded under dual color opsin stimulation that responded to a single excitatory source. A, Examples of whole-cell voltage clamp from two vLGNe neurons, one (top) showing the excitatory postsynaptic responses evoked by repetitive (20 Hz) red light stimulation of retinogeniculate (RG) terminals with ChrimsonR (red traces) and the other (bottom) by blue light stimulation of corticothalamic (CT) terminals with ChR2 (blue traces). Vertical black bars beneath the traces correspond to the timing of photo-stimulation. B-D, Summary graphs comparing the peak responses (B) and PPRs (C,D,) of vLGNe neurons that exhibited convergent input (RG(c) and CT(c) n=11, see Fig. 4) with those that received input from a single source, either retinogeniculate (RG n=7) or corticothalamic (CT n=10). PPRs are expressed as the ratio of the initial EPSC compared to the second (C, EPSC2/EPSC1) or tenth (D, EPSC10/EPSC1). Bars reflect the mean {plus minus} SEM, and symbols individual values. Data for RG(c) and CT(c) are replotted from Fig. 4B-D at 20Hz. Values obtained from neurons that received convergent input were similar to those that received input from a single source.

R1. Use of the Rbp4 reporter line is potentially problematic and bears some unpacking. The authors assert that labelled terminals in this line are derived from L5, which this reviewer challenges on a number of criteria. 1) Reporter lines are notoriously leaky (Prabhakar 2019) and 2) capture cells over development, when a Rbp4 phenotype might be transiently expressed among cells that lack it in maturity. 3) While Rbp4 is restricted to L5 in cortex, it is also expressed subcortically according to Allen ISH data, including sparsely in thalamus and brainstem. Whether these subcortical neurons are labelled in the reporter line is not shown in Fig 1E. 4) The Rbp4 reporter line has been widely used (e.g. by the Clasca lab) and these prior characterizations should be cited.

Author response.

The reviewer raises a somewhat universal concern when using transgenic tools to study neural circuit anatomy and function. Indeed, some transgenic tools, especially intersectional approaches like the Cre-dependent system applied here, suffer from the issue of leaky expression or broad expression, in which expression of a reporter protein or channel rhodopsin is generated in off-target cells (see points 1 and 3 raised by reviewer). The simplest approach to address these common weaknesses of Cre-dependent transgenic tools is to use the reporter protein expression to document what cells in fact generate reporter protein or channel rhodopsin. We have done this in Fig. 1, which illustrates the expression of channel rhodopsin in layer V neurons and their projections into vLGN. In these images you can also appreciate the lack of cellular expression of EYFP-ChR2 in adjacent thalamic regions. According to transcriptomic expression databases, Rpb4, the gene we use to drive Cre expression in layer V cells, is largely not expressed in rodent thalamus but is expressed in just a few select regions of the midbrain (substantia nigra, superior colliculus, periaqueductal gray, and other midline structures) and other regions of the brainstem (including the red nucleus). To our knowledge, most of these structures do not project to the vLGN (Monavarfeshani et all 2017). In the case of the superior collilculus and PAG, projections to the vLGN do in fact exist. However, it is unclear whether these sparse Cre-specific neurons project to vLGN, remain viable in an acute thalamic slice preparation, and if so, whether they provide excitatory or inhibitory input to vLGN. Thus, it remains difficult to ascertain the extent to which they may contribute to the light evoked responses recorded in vLGN. The most parsimonious explanation is that compared to layer V input, these off-target inputs while present are of minimal impact. The reviewer also raises concerns over the transient expression of Rbp4 in these experiments (see point 2 raised by reviewer). In this intersectional approach any expression, even transient, leads to excision of a STOP cassette within the transgenic allele and the subsequent life-long expression of the reporter protein and channel rhodopsin. Thus, we do not think this will be an issue in our case, as we analyzed the expression of channel rhodopsin at ages corresponding to our electrophysiological recordings. Admittedly, we failed to mention the important caveats in using this line as a means to study the function of layer V projections. As requested, the revision includes a discussion about these issues and concerns.

(Results). Several labs make use of the Rpb4-Cre line to visualize or interrogate layer V corticothalamic projections, especially when done in conjunction with a Cre-dependent viral injection approach (Grant et al., 2016; Hoerder et al., 2019; Prasad et al., 2020; Carroll et al., 2021; Hayashi et al., 2021; Casas-Torremocha et al., 2022). While the latter is ideal for achieving cell type specificity, it falls short in providing widespread and sufficient expression in a target structure, a task more readily achieved by using Cre-driver lines. Thus, we chose to take a Cre-driver approach rather than a Cre-viral vector one to maximize expression of ChR2 in an acute thalamic slice preparation. As a result, it opens up the possibility that off-target projections could contribute to our results. According to transcriptomic expression databases (Lein et al. 2007), Rpb4, the gene we use to drive Cre expression in layer V cells, is largely absent in mouse thalamus but is sparsely expressed in select regions of the midbrain (e.g. substantia nigra, superior colliculus, periaqueductal gray) and the brainstem (e.g., red nucleus). To our knowledge, most of these structures do not project to the vLGN (Harrington, 1997; Monavarfeshani et all 2017, Fratzl et al., 2022). However, the superior collilculus appears to project to the vLGN, at least in rodents and cats (Hughes and Chi 1981; Pasquier and Villar 1982; Cosenza and Moore 1984; Fratzl et al., 2021). Thus, it is conceivable that Rbp4 expressing neurons from these regions comprise a fraction of ChR2 containing terminals in vLGN. It is important to note that Rpb4 expression in these off-target regions is very sparse and not well characterized. Moreover, it is unclear if signaling arising from these neurons is excitatory or inhibitory, or whether they remain viable in an acute thalamic slice preparation. Thus, while these off-target inputs are potential contributors, they would appear to represent a small fraction of the excitatory responses evoked by stimulating layer V terminals. The most parsimonious explanation is that compared to the massive layer V input to vLGN, these off-target inputs, while present, are of minimal impact.

R1. In the third paragraph of the introduction, the authors introduce layer 5 as a driver defining higher-order nuclei of dorsal thalamus. Inhibitory cells of the ventral thalamus should be included to strengthen this conceptual framework. A reference to cortico-cortico communication seems here out of place, as the authors note that vLGN includes inhibitory neurons of the ventral thalamus. Why not introduce here other cases of L5 input to inhibitory regions? L5 also inputs to APN (Bokor 2005, Prasad 2020), zona incerta, and the edge of TRN (Carroll 2021), which target higher-order thalamus. Perhaps these inhibitory regions along with vLGNi serve as a gating mechanism or cortico-thalamo-cortical pathways.

Author response. We agree. The revision includes a discussion about how other subcortical inhibitory nuclei receive layer V input thus providing a mechanism for the activation of a trans-thalamic gating signal.

(Discussion)

Indeed, along with vLGNi, other inhibitory nuclei located in caudal prethalamic region receive input from layer V, including the anterior pretectal nucleus, zona incerta, and the outer edge regions of TRN (Bokor et al., 2005; Bartha et al., Bartho et al., 2002; Prasad et al., 2020; Carroll et al., 2022). Since these nuclei project to higher-order thalamic nuclei, input from layer V could serve to trigger a trans-thalamic inhibitory gating signal, perhaps activated during different behavioral states or in a modality specific manner (Halassa and Ascady, 2016; Fratzl et al., 2022).

R1. On the graded effect of EPSP amplitude with increasing stimulus intensity: Was the effect of intensity shown in Fig 2-3 F-G tested statistically (i.e. RM ANOVA)? Such a graded effect is associated with modulator inputs (e.g. Deschenes and Hu 1990), but this is not mentioned in the text. It might fit well in the 2nd and 3rd paragraph of discussion, where the authors list ways in which their synaptic measures deviate from the traditional driver phenotype.

Author response. As noted above (R1. Synaptic stimulation) In the current study, we simply illustrated the relationship between EPSC amplitude and stimulus intensity (Hammer et al., 2014) by generating amplitude by stimulus intensity plots. Our studies and those of the Chen lab reveal that plots showing a gradual or graded increase in EPSP/EPSC amplitude reflect high levels of convergence, while those that are step-like (or all or none response profiles), reflect low levels of convergence.

We discuss how vLGNe have some synaptic features (e.g., high levels of retinal and corticothalamic convergence) that resemble modulator input and does not strictly conform to a driver/modulator framework.

R1. A sentence fragment appears in the intro: “Whereas neurons residing in vLGNi....layer V”.

Author response. We replaced “whereas” with “by contrast”.

R1. In the discussion’s fourth paragraph, the authors state that convergence of drivers is not a common feature of thalamic organization. Is this really known? Provide citations. The authors state that drivers from brainstem and layer 5 converge in POm but no citation is provided (I don’t believe this was shown at the level of single neurons, but rather of the nucleus overall).

Author response. We inadvertently excluded the reference to Groh et al 2014. This reference has been added to the revision. The authors showed both ultrastructural (EM) and electrophysiological (spike probability) evidence indicating that single POM neurons receive convergent input.

Reviewer #2

R2. 1) My main concern is about the overall picture that the manuscript conveys and the underlying supporting data. In the current version the manuscript gives the impression that convergence from retinal and cortical inputs is a common rule in the vLGN while in fact, it appears that only a small fraction of neurons in the vLGN receive both retinal and cortical inputs. Only neurons in the external domain of the vLGN (vLGNe), but not neurons in the internal domain (vLGNi), receive retinal and cortical inputs. Moreover, from those 28 neurons in the vLGNe only 11 receive convergent inputs from both sources. Thus, only about 40% of the neurons in the vLGNe, and none in the vLGNi, actually receive convergent inputs, which is in contrast to the title of the manuscript. Therefore, a more balanced reporting of the data/results is required in the title and main text.

Author response. To address this issue, we made the following changes. The title has been changed from “Convergence of driver like input....” to “Pattern of driver like input...”. We provide additional data and a new figure (Fig. 5) comparing responses of vLGNe neurons that receive convergent input with those receiving input from one source or the other (see above for revised text).

R2.2) Along this line: I think it could be helpful to include the data from the vLGNe neurons that do not receive convergent inputs but only from one source also in figure 4. At the moment figure 4 only shows data/examples from vLGNe neurons with converging inputs. Since the main aim of the figure is to compare RG and CT inputs this makes of course sense. However, I do think that showing also examples from neurons with only retinal and cortical inputs, together with some way of showing the fraction of neurons in each condition (pie chart?), could help the reader to better understand the data.

Author Response. These data have been added to the results section and are summarized in an additional figure (Fig.5, see above).

R2. 3) Similarly, only 10 out of 21 vLGNi neurons show synaptic activity upon light stimulation of the corticothalamic terminals. Where do the other ∼50% of vLGNi neurons get their inputs from? Answering this question is of course beyond the scope of this study, but conveying this information in the main text or figure appears to me to be important.

Author response. The vLGN receives input from a variety of other sources including the midbrain, cerebellum, and brainstem but it remains unclear if these sources provide excitatory or inhibitory input. The percentage of nonresponsive neurons could simply be a consequence of the thalamic slice preparation which could damage or sever connections. These issues are addressed in the revision (see above).

Minor:

R2. 1) Since the study focuses on two subdomains in the vLGN and two input sources it could be helpful to have a small cartoon in the figures showing from where the data is from and which pathway was tested. The figure legends contain this information but adding a small cartoon/inset could make the figure/data even more approachable.

Author response. The addition of a cartoon would make complex figures even denser and detract from the data. Subdomain and input type are stated in the title of the figure legend.

R2. 2) If the authors decide to keep “convergence” in the title it could be helpful to specify the sources (retinal, cortical) already in the title.

Author response. We changed the title (see above).

R2. 3) Figure 3A contains ticks for the optical stimuli, which looks nice. Maybe this could also look nice in Figure 2A?

Author response. These responses were evoked by electrical stimulation. One can see the stimulus artifact in each of the traces. For clarity, we refer to these artifacts in the figure legend.

(Fig. 2 legend). Stimulus artifacts in the traces depict the timing of OT stimulation.
